# A systematic review on trends in using Moodle for teaching and learning

**DOI:** 10.1186/s40594-021-00323-x

**Published:** 2022-01-25

**Authors:** Sithara H. P. W. Gamage, Jennifer R. Ayres, Monica B. Behrend

**Affiliations:** 1 STEM, University of South Australia, University Boulevard, Mawson Lakes, Adelaide, South Australia 5095 Australia; 2 Research & Innovations Services, University of South Australia, University Boulevard, Mawson Lakes, Adelaide, South Australia 5095 Australia

**Keywords:** Moodle, Learning management systems, Education, e-learning, Thematic analysis

## Abstract

**Background:**

The Moodle Learning Management System (LMS) is widely used in online teaching and learning, especially in STEM education. However, educational research on using Moodle is scattered throughout the literature. Therefore, this review aims to summarise this research to assist three sets of stakeholders—educators, researchers, and software developers. It identifies: (a) how and where Moodle has been adopted; (b) what the concerns, trends, and gaps are to lead future research and software development; and (c) innovative and effective methods for improving online teaching and learning.

The review used the 4-step PRISMA-P process to identify 155 suitable journal articles from 104 journals in 55 countries published from January 2015 to June 2021. The database search was conducted with Scopus and Web of Science. Insights into the educational use of Moodle were determined through bibliometric analysis with Vosviewer outputs and thematic analysis.

**Results:**

This review shows that Moodle is mainly used within University STEM disciplines and effectively improves student performance, satisfaction, and engagement. Moodle is increasingly being used as a platform for adaptive and collaborative learning and used to improve online assessments. The use of Moodle is developing rapidly to address academic integrity, ethics, and security issues to enhance speed and navigation, and incorporate artificial intelligence.

**Conclusion:**

More qualitative research is required on the use of Moodle, particularly investigating educators’ perspectives. Further research is also needed on the use of Moodle in non-STEM and non-tertiary disciplines. Further studies need to incorporate educational theories when designing courses using the Moodle platform.

## Introduction

Various learning management systems (LMSs) are available to develop, manage, and distribute digital resources for face-to-face and online teaching. An LMS provides interaction between traditional teaching techniques and digital learning resources, and simultaneously offers students personalised e-learning opportunities (Aljawarneh, 2020). E-learning is an area that has seen considerable growth, particularly since 2020 with the onset of the COVID-19 pandemic, which has limited face-to-face teaching possibilities for many educational institutions globally (Dias et al., 2020; Raza et al., 2021). Educational institutions have had to adapt to restrictions imposed on physical interaction, which have precluded most conventional forms of education, assessment, research, and scientific discourse (Byrnes et al., 2021).

The role of LMSs has gained prominence within the context of STEM (Science, Technology, Engineering, and Mathematics) programs and courses over the last decade through improved access to broadband internet and advancements in online teaching and learning technologies. Many educational institutions have effectively used LMSs and continue to research the effectiveness of using various types of LMSs. Recent studies focussing on STEM education suggest that various LMSs and associated tools increase student engagement, motivation, collaboration (Araya & Collanqui, 2021; Campbell et al., 2020; Hwang, 2020; Jones et al., 2021), performance, retention, and critical thinking (Alkholy et al., 2015; Ardianti et al., 2020; Bernacki et al., 2020; Cadaret & Yates, 2018; Hempel et al., 2020; Oguguo et al., 2021). In addition, LMSs allow STEM educators to track learning outcomes, predict achievements (for early detection of students at risk), and then use the identified information to adapt and modify teaching practices (Dominguez et al., 2016; Hempel et al., 2020; Price et al., 2021; Sergis et al., 2017; Zakaria et al., 2019; Zheng et al., 2019). The future of STEM education can continue to be improved with innovative LMSs and technology-enhanced learning materials (Zhao et al., 2018), such as online laboratories (Henke et al., 2021), online tutorials (Rissanen & Costello, 2021) and virtual reality applications (Christopoulos et al., 2020). A recent systematic review on research trends in STEM education (Li et al., 2020) indicates that ‘learning environments’ which include an LMS is one key area that will continue to evolve.

Currently, 561 LMSs are available worldwide for academic/educational purposes, according to Capterra (2021) an international software review and selection platform. The learning platforms that were most widely used and researched during 2015–2020 include Edmodo, Moodle, MOOC, and Google Classroom (Setiadi et al., 2021). Research on comparisons of various LMSs is rare but some comparisons between LMSs such as Moodle, Sakai, SumTotal, Blackboard, Canvas, and ATutor are available in the literature (Shkoukani, 2019; Xin et al., 2021). According to a recent systematic review on tendencies in the use of LMSs (Altinpulluk & Kesim, 2021), Moodle is the most popular and preferred open-source LMS. Moodle has a high rate of acceptance in the community and in many institutions and has a wide variety of active courses, available in many languages (Al-Ajlan & Zedan, 2008; Sergis et al., 2017). A recent study that determined the effect of LMSs on students’ performance in educational measurement and evaluation recommends that LMSs such as Moodle should be learnt and used by lecturers (Oguguo et al., 2021).

Currently, the world's leading open-source LMS, Moodle (Moodle Project, 2020a), is used by various disciplines within academia, including STEM education. A keyword search of “Moodle” in publications, categorised by discipline area from 2015 to 2021, indicated that more than 60% of publications containing the keyword “Moodle” are in the STEM area. Moodle is a cloud-based LMS and among the top 20 best LMSs based on user experiences in 2018 (Henrick, 2018). The number of Moodle users continues to increase from 78 million in 2015 (Singh, 2015) to over 294 million in 2021(Moodle Project, 2021a)—an increase of over 250%. Although Moodle is becoming increasingly popular, to date, no review has provided information on the use of Moodle across a vast number of disciplines in different educational institutions at different levels of education. This review aims to comprehensively analyse the literature on the adaptation of Moodle as an educational tool over the past 6 years to provide information for three sets of stakeholders—educators, researchers, and software developers. The review addresses two main research questions:

1. Where is Moodle used, adapted, and researched?
2. How is Moodle used in teaching and learning?

## Methods

This systematic review focuses on recent research (January 2015–June 2021) in using Moodle within academic institutions. The review took a multidisciplinary approach to encompass all subjects and levels within academia. To align with the first research question, *Where is Moodle used, adapted, and researched?,* a bibliometric analysis was performed to identify the dissemination of the literature and summarise the bibliometrics of the publications. Then, a thematic analysis was performed to address the second research question, *How is Moodle used in teaching and learning?*.

### Bibliometric analysis

#### PRISMA-P process

This study adopted a strict systematic review protocol that followed the 4-step PRISMA-P process (Moher et al., 2015). This process has the following steps: (1) *Identification* of the relevant literature pertaining to this study, (2) *Screening* using the criteria determined by the authors, (3) *Classification* of the screened articles in a methodical manner using codes and themes predetermined by the authors, and (4) Determining the articles for *inclusion* in this review.

##### Identification

Scopus and Web of Science (WOS) were used to perform the literature search due to their comprehensive journal coverage, ease of keyword searching, accessibility within academia, and popularity within multiple disciplines (Colares et al., 2020; Souza et al., 2019). The term “Moodle” found articles with a wide range of Moodle topics when used in the search databases, while an initial search of Moodle review articles suggested several keywords, such as “Moodle quiz” and “e-learning”. The Scopus search was limited to the selected years with the option of only “Article” or “Review” chosen along with using the title, abstract, and keywords to identify “Moodle” articles. The WOS search was run with “Moodle” and selected all topics in the search parameters. Both database searches were last run on 30 June 2021.

##### Screening

In this phase, literature identified from both database searches was screened to exclude articles that were: (1) published before 2015, (2) written in any language other than English, (3) published but had not been through the peer review process (e.g., conference papers, book chapters, letters), and (4) was not relevant to this review. An individual article's relevance was determined by examining the title, abstract, results, and methods. Any articles that did not fulfil these screening criteria were excluded from this study.

##### Classification

The articles identified and screened were multidisciplinary; therefore, these articles were then classified. Initially, the classification process allocated codes to the journal articles related to the article's research discipline (see Table 1 for codes)—for example, STEM disciplines encompass subject matter of science, technology, engineering, and maths. If more than one discipline was covered in the article, the multidisciplinary (MD) code was used. The articles were then classified into specific subject matter and education levels, including undergraduate, postgraduate, and multi-level. These codes were based on categories of the International Standards Classification for Education (ISCED, 2012). The not-determined (ND) code was used, if needed, for discipline and education level.

**Table.1:** Codes and descriptions for classification of the journal articles included in the review

Discipline		Subject		Education level	
Code	Description	Code	Description	Code	Description
STEM	Science, technology, engineering, maths	S	Science	SC	School, school child
HS	Health sciences	T	Technology	US	Upper secondary
VM	Veterinary medicine	E	Engineering	T	Tertiary
A	Arts	M	Mathematics	UG	Undergraduate
BS	Business studies	A	Accounting	PG	Postgraduate
CS	Computer sciences	L	Languages	TS	Teaching staff
T	Teaching	P	Politics	ML	Multi-level
MD	Multi-disciplinary			ND	Not determined
ND	Not determined				

##### Inclusion

**Fig. 1:**
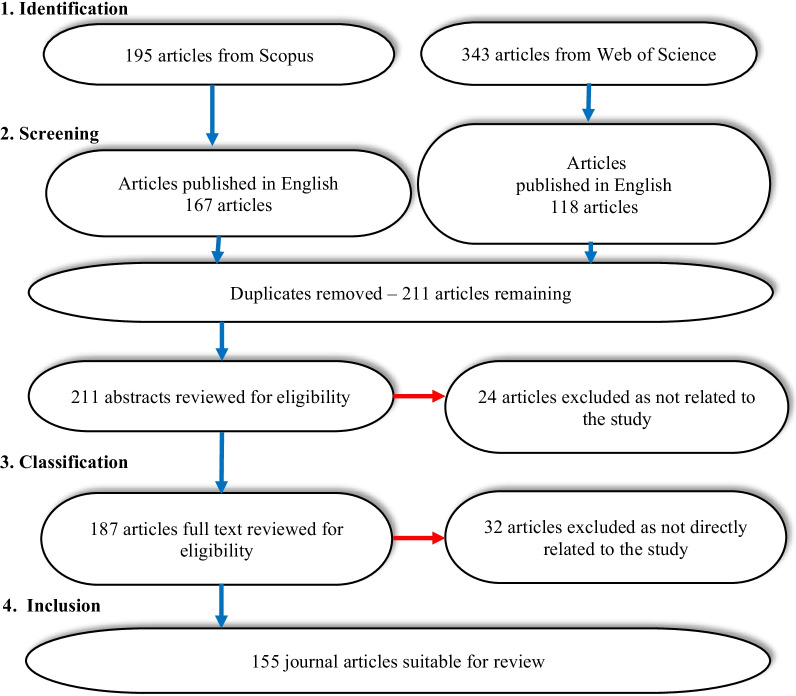
Four-step process for this systematic review

The articles selected for review were limited to Jan 2015–June 2021 and included the word “Moodle” in either the title, abstract, or keywords. The 4-step process applied for selecting the articles included in this review is shown in Fig. 1.

#### Bibliographic analysis

The Vosviewer software, Version 1.6.15, was applied for bibliometric analysis using the Scopus and WOS database search results. Vosviewer is freely available software that automates term identification and constructs bibliometric maps based on network data (Colares et al., 2020; de Souza et al., 2019). The combined downloaded results from Scopus and WOS were used to create a CSV file. The CSV file was updated after the 4-step systematic review protocol process and articles irrelevant to this study were removed from the file. The CSV file was then loaded into Vosviewer to create a co-occurrence map of bibliographic data. The software enables the user to build co-occurrence maps in various areas, such as keywords, journal citation counts, and publication title (van Eck & Waltman, 2020). Bibliometric analysis was conducted on each article, including the year of publication, keywords, journal publication citation count, and the country of publication.

### Thematic analysis (TA)

Following the classification of the included journal articles, further insights and trends within the articles were established by thematic analysis. This process was consistent with Braun and Clarke (2006) thematic analysis (TA) method which identifies and analyses patterns of meanings (themes) in qualitative data. This method can be applied within a range of theoretical frameworks and can be used to analyse almost all forms of qualitative data, both small and large data sets, to address different types of research questions (Clarke & Braun, 2014). The TA used in this review involves the generation of codes and themes. The codes capture features of each paper which have potential relevance to the research questions. The themes were constructed from the coding to capture broader patterns.

To generate the trends identified in the literature, the six-phase Braun and Clarke (2006) method was used as follows:

1. *Familiarisation with the data*: The selected articles were read to become familiar with the topics covered by each article, noting any common concepts covered by each study.
2. *Coding*: Codes were generated for important features relevant to teaching and learning covered by each article (Research Question 2). This coding is not simply a method of data reduction; it is an analytic process.
3. *Searching for themes*: A theme is a coherent and meaningful pattern in the reviewed articles which is relevant to the research questions. The themes were not necessarily in the articles but were constructed. This review constructed eight themes of interest relevant to teaching and learning (Research Question 2).
4. *Reviewing themes*: This step involved reflecting on the themes to tell a story, defining the nature of the theme, and identifying relationships between the themes and different sub-themes within the themes.
5. *Defining and naming themes*: This step involved specifying the ‘essence’ of each theme and constructing an informative name for each theme.
6. *Writing up*: Writing-up involves creating a coherent and persuasive story about the reviewed papers which includes analysis of current and future research.

The themes, sub-themes, and definitions of each theme are shown in Table 2.

**Table.2:** Trend analysis

No	Theme	Sub-theme	Definition of sub-themes
1	Moodle features	Comparing Moodle with other LMSs	Compares Moodle features with other learning management systems
		Moodle tools for student activities	Explains/analyses videos/virtual tours embedded to Moodle, Moodle survey tools, Moodle workshop (for peer assessments), e-portfolios, Moodle lessons, Moodle Quizzes, Moodle discussion forums, and tool 'wiki' as education content
2	Curriculum development	Course design	Discusses online course materials or online course developments
		Design framework	Discusses designing programs (multiple courses) OR upper-level architecture design such as web browser, app server platform development OR teacher training OR quality assurance
		Teachers’ perspectives	Investigates teachers' perspectives, experiences, attitudes of online teaching
3	Learning focus	Adaptive content	Discusses computer-aided interactive content for self-online learning which includes e-learning systems that automatically adapt/generate content based on student preferences and personalities
		Learning styles	Discusses active learning, reflective processing or sensing, intuitive perception or visual, verbal representation or sequential, global understanding
		Critical thinking	Discusses enhancing student thinking ability
		Collaborative learning	Discusses group work and online peer assessment
		Problem/project-based learning	Describes students solving complex problems or online student projects
4	Assessment	Formative assessment	Discusses online formative assessments (non-graded)
		Summative assessment	Discusses online summative assessments (graded)
		Marking and feedback	Discusses grading and providing feedback to formative/summative online assessments
		Online examinations	Discusses online examinations
5	Ethics	Security and privacy issues	Discusses cybersecurity, data protection, user authentications
		Academic integrity issues	Discusses cheating and plagiarisms associated with online exams and assessments
6	Technical developments	Application of Moodle Analytics	Discusses Moodle's in-build analytical tools and how these tools and/or user login data can be used for education research/educational content development
		Software development and adaptation	Discusses new software developments to complement/improve Moodle OR how to use existing software along with Moodle to improve user experiences
7	Research approach and method	Quantitative	Seeks to quantify a phenomenon relevant to online teaching and learning
		Qualitative	Involves descriptive data collection, providing richness of students', teachers', or other stakeholders' thoughts and experiences
		Mixed method	Involves both quantitative and qualitative methods
8	Student success indicators	Student performance	Measures student performance based on their grades
		Student engagement	Measures student engagement of online materials based on whether students engaged in an activity, number of hits (views/attempts) for online activity, how long students have been engaged in a specific activity. Or analyses student behaviours, attitudes, and perceptions of online learning
		Student satisfaction	Investigates student satisfaction and motivation towards online learning

## Results and discussion

The initial database searches identified 538 Moodle-related articles. The literature was then screened for the period Jan 2015–June 2021, journal or review articles only, and articles published in English. This screening reduced the identified articles to 285, 167 from Scopus, and 118 from WOS. These initially screened articles were downloaded from the relevant databases and checked for duplicates. After screening for duplicates, the abstracts from the 211 remaining articles were reviewed, resulting in the elimination of a further 24 articles. The full text of the remaining 187 articles was read, eliminating another 32 articles as they were not directly related to this study. Thus, a total of 155 journal articles were used in this systematic review.

### Bibliometric analysis

#### Journals and citations

**Fig. 2:**
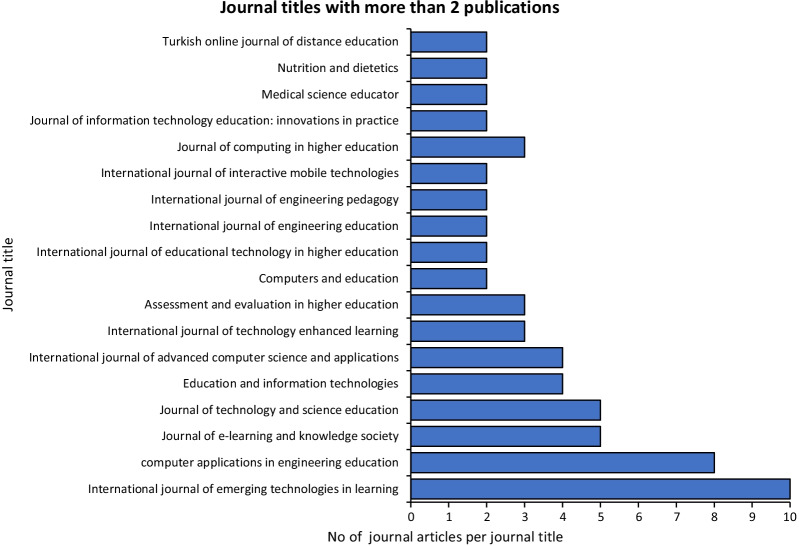
Journal titles that have published more than 2 articles used in this study

Moodle is prevalent in various disciplines, as revealed by 104 journals relevant to this study. Journal titles that published two or more articles are shown in Fig. 2. The journal with the most published Moodle-related articles was *International Journal of Emerging Technologies in Learning* (10 articles), followed by *Computer Application in Engineering Education* (8 articles), and then *Journal of e-Learning & Knowledge Society*, and the *Journal of Technology and Science Education* (5 articles per journal).

Scopus was used for the citation count unless the article was only available in WOS; then, the WOS citation count per article was used. The 155 journal articles reviewed in this study have a combined citation count of 608 with the most cited (71 times) being a review article comparing 17 blended courses using Moodle LMS (Conijn et al., 2017). Total citation counts of the articles by published year were 95 in 2015, 92 in 2016, 270 in 2017, 83 in 2018, 50 in 2019, and 21 in 2020. Of the top 10 cited articles (listed in Table 3), five articles were published in 2017, accounting for 198 citations of the total 270 for that year, with the remaining 72 citations across 24 papers. Of the top 10 authors, four are attributed to the top-cited paper (Conijn et al., 2017). All the top 10 cited authors have articles in the top 10 cited list (Table 4).

**Table.3:** Top 10 cited journal articles

Rank	Article Title	Authors	Journal title	Year	Citation Count
1	Predicting student performance from LMS data: A comparison of 17 blended courses using Moodle LMS	Conijn, Snijders, Kleingeld, & Matzat	IEEE Transactions on Learning Technologies	2017	71
2	An exploration of online behaviour engagement and achievement in flipped classroom supported by learning management system	Wang	Computers and Education	2017	58
3	How learning analytics can early predict under-achieving students in a blended medical education course	Saqr, Fors, & Tedre	Medical Teacher	2017	33
4	Experience of the use of electronic training in the educational process of the Russian higher educational institution	Kamenez et al	International Journal of Engineering & Technology (UAE)	2018	27
5	Development of a problem-based learning model via a virtual learning environment	Phungsuk, Viriyavejakul, & Ratanaolarn	Kasetsart Journal of Social Sciences	2017	25
6	Integrating an online compiler and a plagiarism detection tool into the Moodle distance education system for easy assessment of programming assignments	Kaya & Özel	Computer Applications in Engineering Education	2015	17
7	'I'm not here to learn how to mark someone else's stuff': an investigation of an online peer-to-peer review workshop tool	Wilson, Diao, & Huang	Assessment and Evaluation in Higher Education	2015	15
8	The SIETTE Automatic Assessment Environment	Conejo, Guzmán, & Trella	International Journal of Artificial Intelligence in Education	2016	14
9	Developing a model to assess the success of e-learning systems: evidence from a manufacturing company in transitional economy	Marjanovic, Delić, & Lalic	Information Systems and e-Business Management	2016	13
10	Using log variables in a learning management system to evaluate learning activity using the lens of activity theory	Park & Jo	Assessment and evaluation for continuing in higher learning	2017	11

**Table.4:** Top 10 cited authors

Author	No. documents	Citations	Journal Title
Conijn R	1	71	Predicting student performance from LMS data: A comparison of 17 blended courses using Moodle LMS
Kleingeld A	1	71	
Matzat U	1	71	
Snijders C	1	71	
Wang F. H	2	60	On the relationships between behaviors and achievement in technology-mediated flipped classrooms: A two-phase online behavioral PLS-SEM model
			An exploration of online behaviour engagement and achievement in flipped classroom supported by learning management system
Fors U	1	33	How learning analytics can early predict under-achieving students in a blended medical education course
Saqr M	1	33	
Tedre M	1	33	
Smirnova Z. V	3	39	Experience of the use of electronic training in the educational process of the Russian higher educational institution
			The organization of the test control of students' knowledge in a virtual learning environment Moodle
			Assessment tools in e-learning Moodle
Vaganova O.I	3	39	

#### Author-affiliated countries

**Fig. 3:**
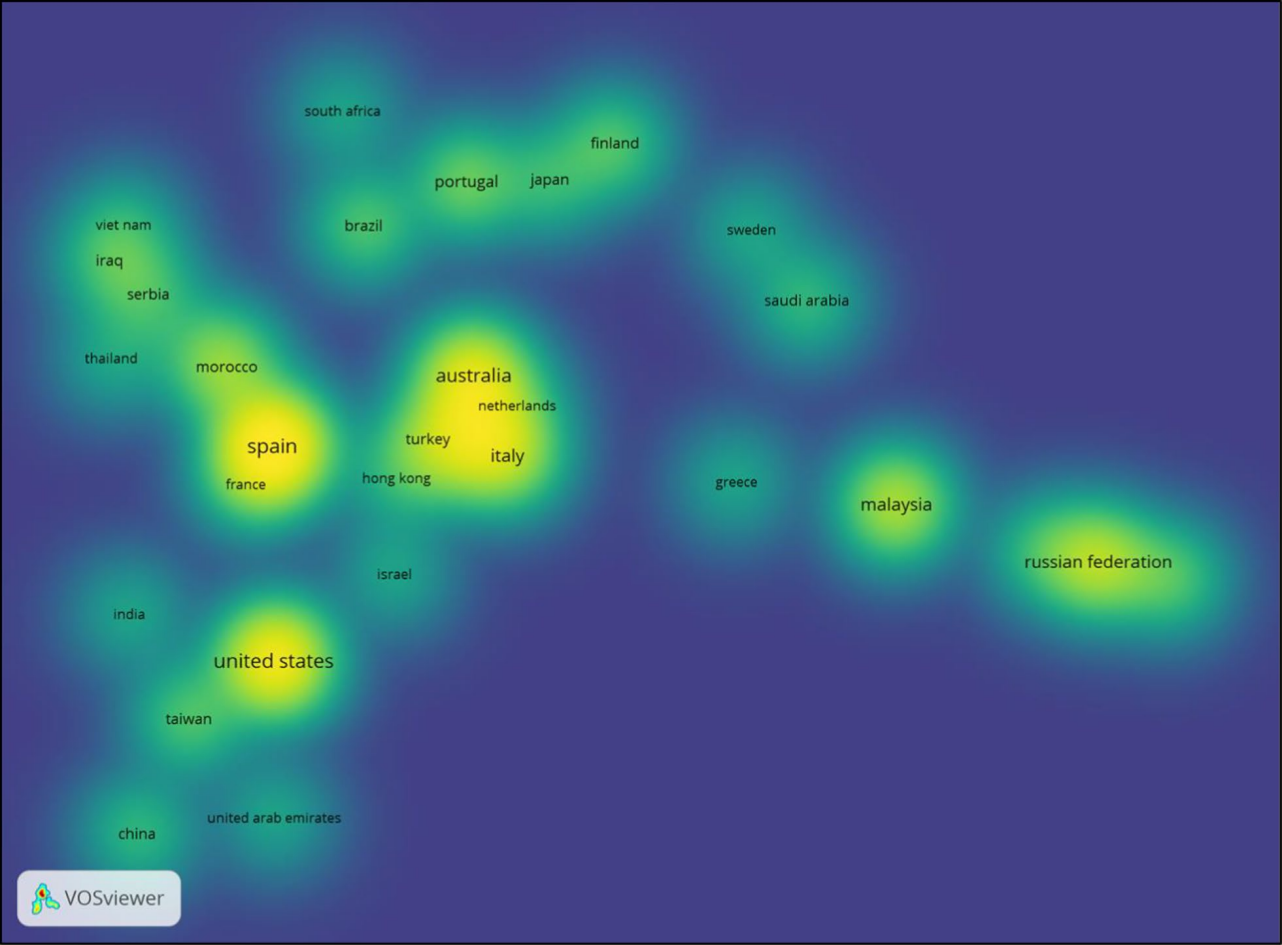
Density map showing countries contributing to more than 2 publications

Vosviewer has the facility to produce a density map of co-occurrences in countries (van Eck & Waltman, 2020). Figure 3 shows the density map of countries publishing more than two articles. Fifty-five countries contributed research to the 155 articles, with 37 countries publishing more than two papers. The higher the count of publications, the brighter the yellow, with Spain contributing 17 articles, the United States of America (USA) 14, Australia 12, the Russian Federation 10, Malaysia 8, Italy 7, and Portugal 5 articles. The software positions the countries with a similar number of articles published close to each other. Therefore, Vosviewer provides the reader with an instantaneous pictorial result of countries publishing Moodle articles.

#### Popular keywords

The keywords from the 155 articles were analysed in Vosviewer. In total, 926 keywords were used, of which 154 were used three times or more. Table 5 shows the top 10 keywords. The most occurring keyword was Moodle (61), followed by e-learning (31), teaching (26), and education (25), and learning management system (25).

**Table.5:** Top 10 popular keywords

Rank	Keyword	No of times used
1	Moodle	61
2	E learning	31
3	Teaching	26
4	Learning management system	25
5	Education	25
6	Students	21
7	Human	18
8	Assessment	14
9	Female	11
10	Male	10

**Fig. 4:**
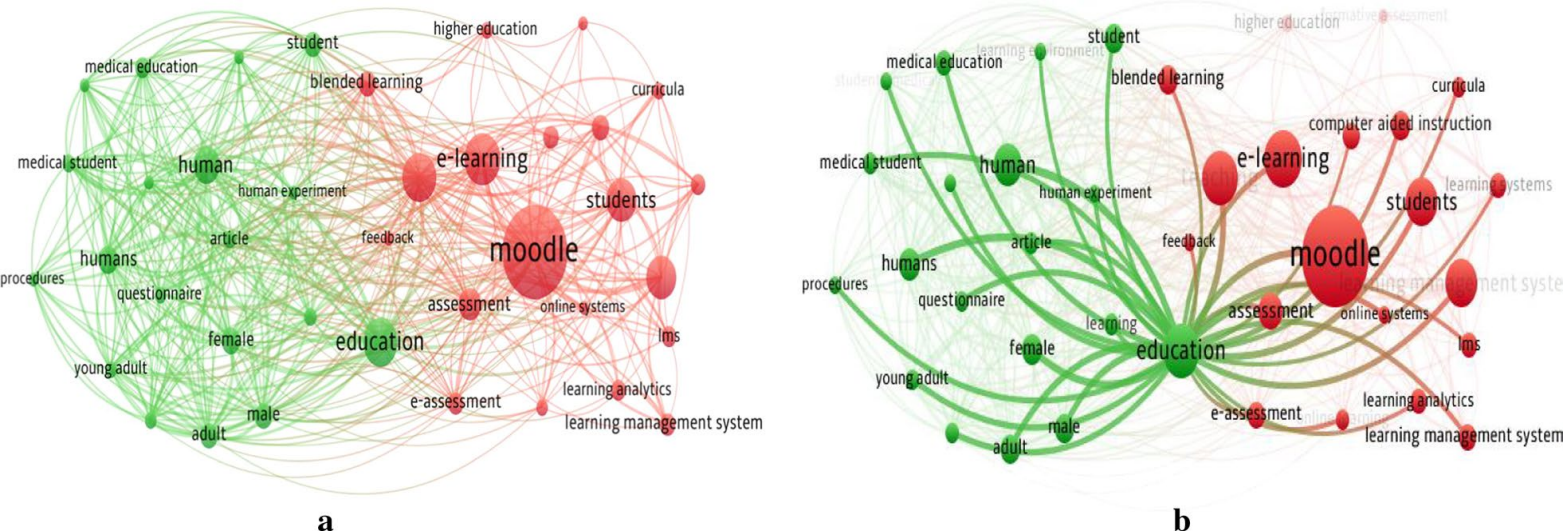
Vosviewer cluster graphic of keyword results: **a** Keywords with more than 3 uses, **b** The links highlighted when the word ‘education’ is highlighted

Along with the ability to extract the top keywords used within the articles, Vosviewer produced cluster graphics of keywords. Figure 4a shows the cluster graphic of keywords of more than three uses or a higher density with a larger marker on the graphic; hence, the most significant markers are Moodle, e-Learning, and Education. The map also has the feature to zoom in and out, showing more keywords and highlighting the most occurring keywords. Figure 4b shows the option in Vosviewer to see the links that connect the keywords within the articles (in this instance, Education was highlighted). The keywords associated with Education in the 155 articles (with more than 3 occurrences) can be seen with linked keywords, such as Moodle, Student, and e-Learning. All keywords can be highlighted individually for associations to be seen.

#### Discipline and education level of studies

**Fig. 5:**
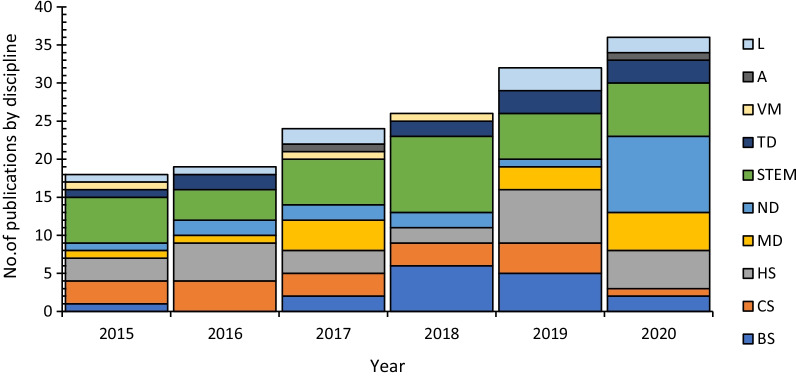
No of publications by discipline 2015–2020

Research into Moodle assessments is being published in many different subject areas, such as science, technology, engineering, and maths (STEM), health sciences (HS), and veterinary medicine (VM). Figure 5 shows the number of publications per full year (2015–2020) and the articles' discipline.

L—languages, A—arts, VM, veterinary medicine, TD—teaching degree, STEM—science, technology, engineering and maths, ND—not determined, MD—multi-discipline, HS—health sciences, CS—computer science, BS—business studies.

The number of total publications was lowest in 2015 and 2016 with 18 and 19 publications, respectively. This number increased each year after that: 2017 (n = 24), 2018 (n = 26), 2019 (n = 32), and 2020 (n = 36). The two main disciplines throughout this publication period were STEM and HS. The STEM discipline contained various subjects, with most being engineering (civil) and science (i.e., physics and chemistry). HS subjects published include nursing, medical practice, and dentistry. Some articles that did not fit into a particular discipline (ND) covered various subjects, such as security issues identified within e-learning or articles that deal with databases (Chaparro-Peláez et al., 2019; Mudiyanselage & Pan, 2020).

Of the 155 articles, 116 articles evaluated Moodle within a university setting, with 112 at undergraduate (UG) level, nine postgraduate (PG), and seven articles examined at both UG and PG courses. School-age students (S) were the focus in six studies, teaching staff (T) in four articles, and S and T in two articles. A total of 31 articles did not determine (ND) the level of education for the study or were not focused on individuals but rather systems (Chafiq et al., 2018; Conejo et al., 2016).

### Thematic analysis (TA)

**Fig. 6:**
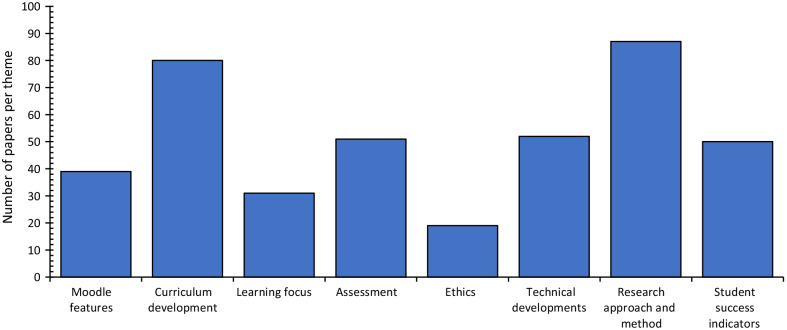
Frequency of articles describing each theme

**Fig. 7:**
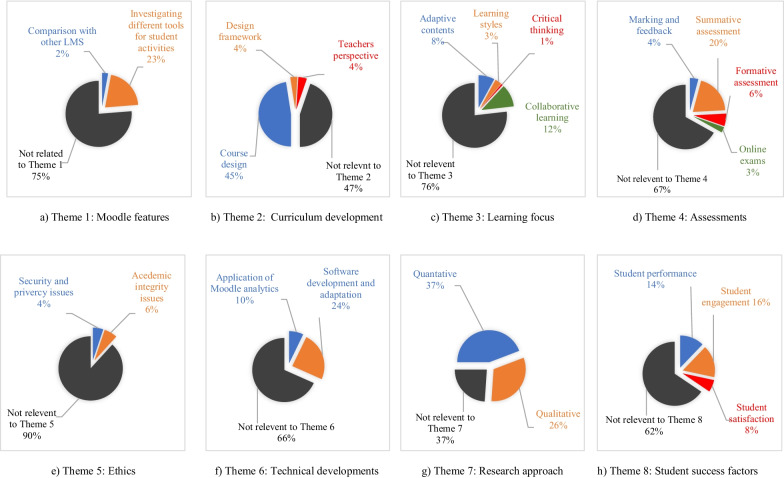
Percentage of research papers related to each theme and sub-theme

The trends demonstrated in the research articles are categorised into eight main themes (see Table 2). Theme 1 compares various Moodle features explained in the study. Themes 2 to 4 highlight the trends in pedagogy, which include curriculum development, learning, and assessment processes in e-learning. Theme 5 analyses ethical aspects of e-learning, and Theme 6 highlights trends in new software development aiming to improve e-learning, particularly Moodle. Themes 7 and 8 provide an overview of research approaches, methods, and common student success indicators. Figure 6 shows the number of papers that discuss each of the eight themes, although several papers discuss multiple themes. Figure 7 shows the percentage of papers related to each theme and sub-theme.

#### Theme 1: Moodle features

**Fig. 8:**
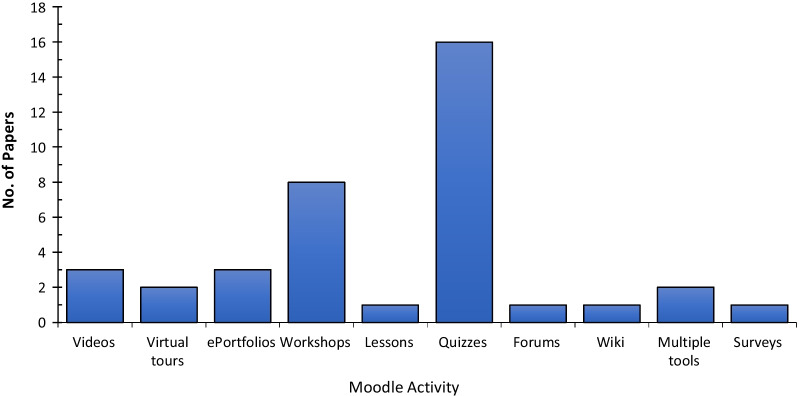
Number of articles reporting research on each Moodle activity

Of the reviewed articles discussing Moodle LMS, 23% discuss Moodle ‘Activities’. An activity, a general name for a group of Moodle features, is usually something that a student will engage in and that interacts with other students or the teacher. The activities identified included: Moodle quizzes, forums, workshops, lessons, wikis, and surveys. Of these, Moodle quizzes and workshops were the most prevalent, with 16 and eight articles, respectively (see Fig. 8). Some activities, such as videos, virtual tours, e-portfolios, are external tools easily embedded into the Moodle system.

None of the articles discussed Moodle activities, such as Choice, Database, Feedback, Glossary, H5P activity, or SCORM (for course content). One study (Sánchez et al., 2015) recommends Moodle's “Survey” tool for anonymous surveys, yet if this tool is used along with Moodle’s “Group” option, the users can determine who responds to the survey. Therefore, the “Feedback” activity is a better anonymous survey tool than the “Survey” activity.

Except for Shkoukani (2019), who analysed features for the 20 most popular LMSs in 2018, few studies compare Moodle with other LMSs. Only 2% of papers analysed in this study have compared Moodle with other LMSs, and they only compared Moodle with Blackboard or Canvas (Aljawarneh, 2020; Shdaifat & Obeidallah, 2019). Further analysis between LMSs focusing on features, integration, cost, and security are pivotal for e-learning success.

#### Theme 2: curriculum development

In 53% of the reviewed articles, LMS Moodle was used for curriculum development, including implementing learning modules and assessments for blended and online courses. While about half of the articles (45%) explain how this can be used at the course level (e.g., Awofeso et al., 2016; Brateanu et al., 2019; Chootongchai & Songkram, 2018), 4% of the articles explain how this can be used for framework design (multiple courses to achieve program objectives) (e.g., Kouis et al., 2020; Saleh & Salama, 2018; Smolyaninova & Bezyzvestnykh, 2019).

Educators bear responsibility for ensuring optimal tools are utilised for the most effective computerised assessment that enables students and teachers to address or avoid assessment-related problems (Marczak et al., 2016). However, only 4% of papers analyse the teachers’ perspectives of using Moodle (Babo & Suhonen, 2018; Badia et al., 2019; García-Martín & García-Sánchez, 2020; Jackson, 2017; Marczak et al., 2016; Valero & Cárdenas, 2017). Badia et al. (2019) conducted a study using 132 teachers across 43 schools indicated further research should be conducted on: Why do only certain Moodle activities positively impact learning outcomes? What can technological designers and teachers do to improve the level of learning outcomes achieved through the use of Moodle activities?

Of the 155 articles reviewed, only eight used educational theoretical frameworks for their research and development (see Table 6). According to the studies shown in Table 6, online assessments can be theorised using Classical Test Theory (CTT) and Item Response Theory (IRT). Online content development, particularly adaptive content, can be theorised using Computer Adaptive Testing (CAT), the Technology Acceptance Model (TAM), Merrill's problem-centric framework, and Self-determination theory. The DeLone and McLean Information Systems (IS) theories can be used to measure the effectiveness of advanced online materials and for the implementation of e-learning systems.

**Table.6:** Education theories used in research and development

Theory	Description	References (description cited in)
Moore’s Transactional Distance theory	This theory was developed in the 1980s to investigate two variables: students’ autonomy and the distance between students and teachers. This theory mainly describes the learner and the educator/teacher relationship	Abuhassna & Yahaya, (2018)
Item Response Theory (IRT)	The IRT was initially developed in the 1940s and intended to measure a variable of interest. (e.g., ‘assessment’ – where the ability to solve equations can be measured)	Azevedo et al., (2019); Conejo et al., (2016)
Classical Test Theory (CTT)	The CTT dates to the beginning of the twentieth century, with its origin in psychology. CTT is concentrated on the difficulty and the discrimination of the questions	Azevedo et al., (2019); Conejo et al., (2016)
Technology Acceptance Model (TAM)	The TAM originated in the late 1980s from the Theory of Reasoned Action (TRA) and has been broadly used to predict and explain human behaviour in various domains. According to the TAM, students' beliefs about the technological system determine their success in technology-based activities	Basol & Balgalmis, (2016)
Self-determination theory	The self-determination theory continuously developed in the 1980s through to the 2000 focuses on personality development, self-motivation and behavioural change. This is an approach to motivate people and change their personalities through discovering new thoughts, mastering new skills and satisfying their requirements	Chemsi et al., (2020)
Computer adaptive testing (CAT)	The CAT tries to modify the selection of questions to maximize the information obtained. It can be proved that this condition is equivalent to the selection of the question whose difficulty is closer to the currently estimated student knowledge	Conejo et al., (2016)
Quasi‐experimental research methodology	Quasi-experimental research methodology has two equivalent groups and no control group in which successive measures have been conducted but always within the intervention, that is, under the influence of the independent variable	Gaona, et al., (2018)
The DeLone & McLean IS success model	This model was first introduced in 1992, in which the system quality, information quality, use, user satisfaction, individual impact, and organizational impact are distinct, but related dimensions of IS (Information systems) success	Marjanovic et al., (2016)
Activity theory	Activity theory has been an interdisciplinary approach to human science and an evolving theoretical framework through several generations and multiple perspectives	Park & Jo, (2017)
Merrill's problem-centric framework	This framework is to engage with students cognitively, emotionally, and behaviourally. This framework was proposed in 2013, designing principles for “e3 learning” (effective, efficient, and engaging learning)	Wang, (2019)

#### Theme 3: learning focus

Adaptive, collaborative, or problem-based content developments were discussed in 20% of the articles, with only 4% considering learning styles and critical thinking.

##### Adaptive learning

LMSs provide large data databases and fast access to a systematic analysis of information. Therefore, designing adaptive or self-learning modules and automatic assessments which adapt to the learner’s preferences has become much easier. Of the articles reviewed, 8% either demonstrate or improve automated content. The areas addressed within these articles were randomly generated tests, questions with multiple possible answers, automated marking systems and rubrics, provision of positive and motivational automatic summative and formative feedback, auto-adaptive content for learners with diverse backgrounds, interactive content, self-assessed quiz and multimedia books for instructional design (Azevedo et al., 2019; Brateanu et al., 2019; Gutiérrez et al., 2016; Ljubimova et al., 2015; Paiva et al., 2015).

Further research has investigated integrating instructional design theories, psychological elements, and learning theories into adaptive learning (Abuhassna & Yahaya, 2018; Conejo et al., 2016; Saleh & Salama, 2018). Tlili et al. (2019) conducted a study that aimed to model the learners’ personalities using a learning analytics approach called intelligent Moodle (iMoodle), with results compared to the traditional method of modelling learners' personalities using questionnaires (Tlili et al., 2019). A further study investigated automatic detection of learning styles by analysing student learning behaviour by constructing a mathematical model (Xiao & Rahman, 2017). Further research has been suggested in the areas of exploring the extent to which automatic feedback encourages positive motivational beliefs and self‐esteem among students (Gaona, et al., 2018), improving real-time adaptation learning modules, intelligent non-human tutoring, and using educational data mining techniques to investigate and predict students' attitude to learning.

##### Collaborative learning

Collaborative learning was discussed by 12% of the reviewed articles. Of these, a number focused on Moodle's peer assessment tool “workshop” and demonstrated how to use “workshop” to allow students to mark their fellow students’ work and reduce the marking load for teaching staff (ArchMiller et al., 2017; Slee & Jacobs, 2017; Strang, 2015). Peer review and feedback were generally accepted as helping to develop students’ meta-cognitive skills relating to critical reflection (Wilson et al., 2015). However, qualitative studies show that students and staff have divided opinions regarding the “workshop” tool for peer assessment (Divjak & Maretić, 2017; Dolezal et al., 2018; Wilson et al., 2015). While students agree with a limited number of peer assessments, staff experience an increase or no decrease in their marking workload (Wilson et al., 2015). However, peer assessments using “workshop” are still time-consuming for both the teacher and students and could lose their charm if they are overused (Dolezal et al., 2018). In studies that have used peer assessments to allow students to grade their peers, some students reported the peer assessment method as “unfair and “unprofessional” (Divjak & Maretić, 2017; Dolezal et al., 2018; Wilson et al., 2015). The “workshop” tool in Moodle does not have a built-in measure for peer assessment validity. One study which addressed the concern of students’ validity contributing to marking assignments reported that the grades were consistent with what faculty expected based on *t* tests and reliability estimates (Strang, 2015).

The Moodle activity “Forum” can be used to improve problem-based learning via group projects (Awofeso et al., 2016). “Forums” allowed students to maintain much more direct contact when they were not in the class and made it easier for students to meet and work on their projects even though they were in different places (Marti et al., 2015; Phungsuk et al., 2017). A further study reported that online learning systems positively influenced students' thinking and innovation skills (Chootongchai & Songkram, 2018).

##### Learning styles

Of the identified articles, 3% investigated learning styles—namely, Active vs Reflective, Sensitive vs Intuitive, Visual vs Verbal, Sequential vs Global—when implementing e-course content (Kouis et al., 2020; Ljubimova et al., 2015; Xiao & Rahman, 2017). These studies have shown that students' independent work can be guided through interactive technology, and these teaching methods would eliminate students' passivity in the classroom and enhance their cognitive activity. While some studies have proposed automatic detection of learning styles by analysing student's learning behaviour through mathematical models (Xiao & Rahman, 2017), other studies have developed simpler matrix systems that would allow the teacher to carry out a manual selection of tools for Moodle Learning after considering student's learning styles (Ljubimova et al., 2015; Meza-Fernández & Sepúlveda-Sariego, 2017; Xiao & Rahman, 2017). However, identifying students' learning styles to maintain assessment quality needs further investigation (Meza-Fernández & Sepúlveda-Sariego, 2017).

#### Theme 4: assessment

A third (33%) of the reviewed papers focused on assessment including summative and formative assessment, online exams, marking, and feedback (Adesemowo et al., 2016; Albano & Dello Iacono, 2019; Basol & Balgalmis, 2016; George-Williams et al., 2019). Moodle can create large data pools of various questions, including multiple-choice, open answer, generative questions, and complex tasks (Conejo et al., 2016). Nevertheless, most papers focused on summative assessment based on Moodle quizzes investigating both teachers’ and students’ opinions when implementing multiple-choice questions (Babo & Suhonen, 2018; Cakiroglu et al., 2017; Dimic et al., 2018; McKenzie & Roodenburg, 2017; Shdaifat & Obeidallah, 2019). According to a 5-year study, the ‘luck’ factor associated with multiple-choice questions is fair (Babo et al., 2020). Studies that have investigated the students' point of view indicate that the students agree that Moodle is easy to use and complements teaching, although most students still prefer classical assessment techniques (Cakiroglu et al., 2017; McVey, 2016; Popovic et al., 2018). However, one study found no direct relationships between students' preferences and academic performance (Cakiroglu et al., 2017).

Some studies which focused on the assessment process investigated the usefulness of the online environment for instructors to organise assessments, the usefulness of giving responsibilities to students during assessment (mainly via peer assessments), and using Moodle statistics and analytics to evaluate and improve the quality assessment process (Cakiroglu et al., 2017; Gamage et al., 2019; Hussain & Jaeger, 2018).

##### Marking and feedback

Four percent of reviewed articles focused on improving and streamlining the marking and feedback processes for both students and teachers. These studies indicate that online marking systems associated with Moodle lower the long-term costs, increase the speed of providing feedback, provide greater flexibility with respect to location and timing and reduce the space required to manage the assessment process (García López & García Mazarío, 2016; Koneru, 2017; Villa et al., 2018). A study with 57 academics conducted at Monash University, Australia, highlighted Moodle's reliability, and improved impartiality of the assessment process (George-Williams et al., 2019). The study concluded that this impartiality is generally achieved through the removal of personal, academic judgment, which results in more reliable, consistent marking practices.

#### Theme 5: ethics

The reviewed articles investigated two strands of ethics: (1) ethics relating to users' data security and privacy, and (2) academic integrity. While 4% of all reviewed articles highlighted security and privacy concerns, 6% of the articles discussed academic integrity issues caused by the increased use of LMSs for assessment purposes. Although personal data protection has legal compliance, such as the policies in the European Union and the Privacy Act 1988 in Australia, several articles discussed the privacy concerns of cloud-based services. The use of cloud-based services has resulted in teaching materials being stolen, and instructors' or administrators' credentials being compromised (Daniels & Iwago, 2017; Kiennert et al., 2019; Mudiyanselage & Pan, 2020).

Two re-occurring academic integrity issues associated with online assessments were highlighted: students plagiarising and students using third parties to complete assignments (Amoako & Osunmakinde, 2020; Guillén-Gámez & García-Magariño, 2015). Although instances of these two integrity problems occur in traditional teaching and learning methods, face-to-face invigilated exam environments can help minimise the effect of these issues. One alternative to invigilated exams is online quizzes which have become popular due to their ability to automate marking. However, cheating cannot be controlled unless it is held in an invigilated room. Several studies attempted to address this issue by introducing new software and analytical tools to detect academic misconduct. These tools include: limiting IP range for the users during online exams (Adesemowo et al., 2016); using timestamps and data processing techniques to identify unauthorised users (Genci, 2014); using facial verification software (Guillén-Gámez & García-Magariño, 2015) and using plagiarism detection software (Adesemowo et al., 2016; Genci, 2014; Guillén-Gámez & García-Magariño, 2015; Kaya & Özel, 2015).

#### Theme 6: technical developments.

##### Application of Moodle analytics

Online LMSs make it more manageable to gather and analyse students’ data. Ten percent of the articles reviewed discussed the in-built statistical tools such as the facility index and discrimination index along with the databases available in LMSs for the use of educational and research purposes (Fenu et al., 2017; Gamage et al., 2019; Monllaó Olivé et al., 2020). The articles used data mining and statistical tools to measure and analyse student engagement, student satisfaction, and online courses' performance. Analysing the tools available would be beneficial for monitoring student retention rates (Monllaó Olivé et al., 2020), identifying underachieving students (Saqr et al., 2017), predicting students' trends and attitudes, and accreditation purposes (El Tantawi et al., 2015; Saleh & Salama, 2018; Strang, 2016). Data and analytics tools may also be used to automate personality assessments and create intelligent (adaptive) learning platforms (Tlili et al., 2019).

##### Software development and adaptation

This review found that 24% of the articles discussed or evaluated software development and adaptations, including the use of existing software to improve the learning experience within Moodle. Software applications that can be integrated into Moodle include:

- Apple's Siri and Google's GRScloud-based speech recognition for language learning (Daniels & Iwago, 2017).
- OpenIRS-UCM (García López & García Mazarío, 2016), Kahoot, Poll-Everywhere and Zappar (Hsiung, 2018) which are tools for interactive polling.

The ever-increasing number of new software/Add-Ins available for Moodle is indicative of the interest of software developers and researchers to improve the useability of Moodle for online teaching and education. Course developers utilise plug-ins to assist with automatic essay marking, randomising questions, and identifying ineffective questions (Koneru, 2017; Schweighofer et al., 2019; Villa et al., 2018). Table 7 lists several software applications that can be integrated into LMSs and, in particular, Moodle.

**Table 7:** Software applications to enhance existing LMS

**Name of advancement**	**Key purpose/ function**	**References**	**Advancements in:**									
			Security	Assessment process	Cognitive learning skills	Collaborative e-learning	Learning Analytics	Applications of artificial intelligence	Maintaining academic integrity	Providing feedback	Navigation	Speed & response time
SEB – Safe exam browser	Improves the security of e-assessments/ exams	Adesemowo et al., (2016)	X	X								
Inno Ed Tools	for assessing students' thinking and innovation skills	Chootongchai & Songkram, (2018)			X							
SIETTE	For creating large item pools of different types of questions, including multiple-choice, open answer, generative questions, and complex tasks	Conejo et al., (2016)		X								
Under development	An analytic tool for user interface evaluation	Fenu et al., (2017)					X					X
LAMS and CMS Alfresco	Intellectual mechanisms for managing personalized learning in an educational environment	Finogeev et al., (2020)			X			X				
Under development	Automatic argument assessment tool which identifies arguments and provides recommendations to improve students’ writing.	Gray et al., (2018)						X				
Smowl	Facial authentication system	Guillén-Gámez & García-Magariño, (2015); Guillen-Gamez et al., (2015)	X									
Sharable Auto-Adaptive Learning Object (SALO)	Realtime adaptation software for learning content	Gutiérrez et al., (2016)		X								
Moss source code plagiarism detection tool	A source code plagiarism detection tool for programming courses	Kaya & Özel, (2015)		X					X			
e TeSLA project (Adaptive Trust e-Assessment System)	For data protection in e-learning platforms	Kiennert et al., (2019)	X									
LiquiZ	An assessment engine that provides advanced question types that allow teachers to ask questions that can currently only be asked in one-on-one demonstration. (Targeted toward STEM subjects and particularly advantageous in math or science subjects)	Kruger et al., (2015)		X								
Socrative	A collaborative learning tool to improve and streamline feedback processes for both students and teachers.	García López & García Mazarío, (2016)				X				X		
Under development	Intelligent system for collaborative e-learning	(Matazi et al., 2018)				X		X				
PeerWise	For promoting engagements with peers - engaging students in authoring, answering, and evaluating MCQs as a formative assessment	McKenzie & Roodenburg, (2017)			X	X						
STACK	Enables mathematical input to be evaluated by a symbolic analysis software	Neitola, (2019)		X						X		
UVLEQoC	A virtual environment that could provide the adaptations to the users’ context	Nunes et al., (2015)			X			X				X
Under developments	Interactive and multimedia content associated with a system for computer-aided assessment	Paiva et al., (2015)		X								
ASHuR	A method for automatic text summarisation to evaluate a summary built by humans	(Ramírez-Noriega et al., 2018)						X	X			
MoodleNFC	Facilitates scanning IDs for recordkeeping and grading purposes	Ross, (2017)	X								X	X
WIRISquizzes	A powerful calculator with a friendly math editor which also allows self-evaluation	Sancho‐Vinuesa, et al., (2018)		X								
The PHP application	A self-assessment tool	Schweighofer, et al., (2019)		X								X
LaMoo	Innovation in teaching technical drawing	Villa et al., (2018)		X								X

To date (June 2021), Moodle has 1753 available plug-ins that can add new functions that improve administration, assessment, collaboration, communication, content and the interface (Moodle Project, 2020b). The Moodle statistics for 2019 show that the most popular plug-ins (based on the number of downloads) were communication and content plug-ins, such as Moove, BigBlueBN, Adaptable, H5P, and Eguru (Moodle Project, 2020b). The articles in this review covering Jan 2015–June 2021 show that most reported advancements in new software developments for Moodle relate to improving assessment processes. The development advancements include improving the security of the assessment processes (Adesemowo et al., 2016; Kaya & Özel, 2015), improving the mechanisms to generate quiz questions, and improving feedback and response time (Conejo et al., 2016; Kruger et al., 2015). Security improvements include, but are not limited to, improving user data verification (Amoako & Osunmakinde, 2020), facial recognition (Guillén-Gámez & García-Magariño, 2015), limiting IP range (Adesemowo et al., 2016), and scanning students IDs (Ross, 2017). Daniels and Iwago (2017) also reported on integrating Google speech recognition for speech assessments. Improving students cognitive, innovative, and collaborative learning skills were a key area of development in some reported studies (Chootongchai & Songkram, 2018; Finogeev et al., 2020; García López & García Mazarío, 2016), along with the improvement of user interface evaluation (Fenu et al., 2017).

Artificial intelligence tools are an increasing area of research which investigates intellectual mechanisms for managing personalised learning. Gray et al. (2018) reported on the software developments that aid students in their report writing and allow arguments, justification, and conclusions to be formed without any human input. Software development also encompasses the ability to direct students to relevant content and assessments after automatic analysis of the students' behaviour (Finogeev et al., 2020) and can also evaluate summaries written by students using information available on websites and online repositories (Ramírez-Noriega et al., 2018). As software advancements to assist students with their assignments are increasing, so is plagiarism. Plagiarism detection systems are successfully integrated into Moodle with plug-ins, such as Urkund, Turnitin, Plagiarisma, and SafeAssign which can detect textual plagiarism. Source code detection software for programming courses are under development (Kaya & Özel, 2015).

Despite advances in software and technology for e-learning and online LMSs, numerous fundamental gaps/drawbacks still exist, with the majority on technical issues (Adesemowo et al., 2016; Marczak et al., 2016; Rachman‐Elbaum, et al, 2017), such as server/browser response times, lag time in resolving technical issues, lack of equipment available to students and the possible high cost associated with the initial development of programs (Chang Chan et al., 2019; El Tantawi et al., 2015; Marczak et al., 2016; Zamalia & Porter, 2016).

#### Theme 7: research approach and methods

**Fig. 9:**
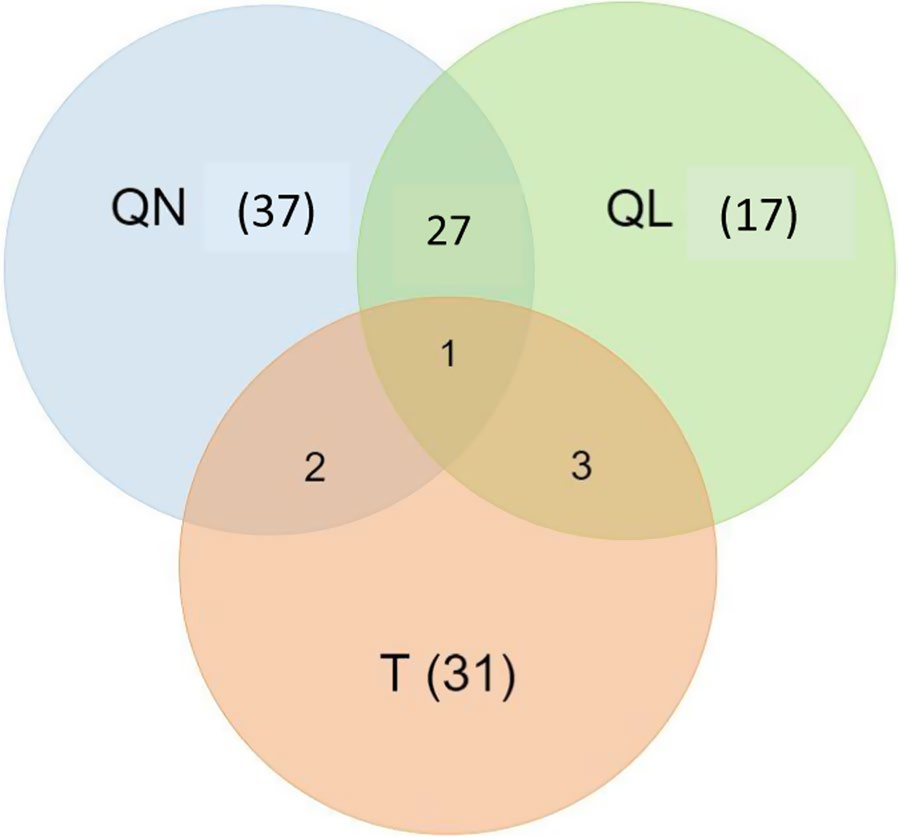
Venn diagram for QN (quantitative), QL (qualitative), and T (technical) types of research

The research approaches used are categorised into quantitative analysis, qualitative analysis, mixed methods, technical and other. Of the 155 articles reviewed, 67 papers used a quantitative (QN) research approach which aimed to quantify a phenomenon relevant to online teaching and learning (see Fig. 9). Forty-eight papers used a qualitative (QL) research approach which involved descriptive data collection, student, teacher, or other stakeholder thoughts and experiences; 28 papers used mixed methods—both qualitative and quantitative approaches; 37 papers discussed technical (T) components of LMS and included new software development and framework design; and, 37 papers were categorised as “other”, namely, research that did not fall into the above three categories, e.g., applications of existing LMSs and tools, reviewing/comparing existing LMSs or tools.

Qualitative research studies in this review evaluated mainly the students’ perspective: their preferences, perceptions, satisfaction, and attitudes towards online learning, including the online tools being utilised (Botelho et al., 2020; Cakiroglu et al., 2017; García-Martín & García-Sánchez, 2020; Tsai & Tang, 2017). Only two research studies focused exclusively on teacher opinions, perceptions, and experiences in e-assessment, Moodle activities, and their learning impacts (Babo & Suhonen, 2018; Badia et al., 2019). Four articles reported on both student and teacher perspectives and discussed attitudes towards summative and formative assessments and flexibility in e-learning (Jackson, 2017; Kamenez et al., 2018; Marczak et al., 2016; Valero & Cárdenas, 2017). Jackson (2017) reported that Moodle is a technology that enables creativity among teachers and recommended that management incorporate training programs of LMSs for both teachers and students into their strategic plans.

#### Theme 8: student success factors

The qualitative, quantitative, and mixed methods research have common indexes used as student success indicators, namely, student performance, engagement, and satisfaction indicators (as described in Table 2). Figure 7h shows the articles that discussed student success factors with 14% using student performance, 16% student engagement, and 8% student satisfaction. Student performance and engagement are mainly found in quantitative research, whereas student satisfaction indicators are found in qualitative research. Qualitative research measuring student satisfaction are fewer than quantitative research analysing student performance and engagement.

## Conclusion

This comprehensive systematic review on Moodle use for online teaching and learning covers a wide range of educational institutions. The review identifies methods used and developments over the last 6 years published in 155 journal articles across 104 journals over 55 countries and 10 disciplines. The findings have been summarised bibliographically and thematically where appropriate, providing vital information to educators, researchers, and software developers. The critical limitation of this review is that only Scopus and Web of Science databases were used for the search, and papers that are not covered by either database are not included in this analysis.

The bibliographic analysis identified Moodle as a well-established and advanced learning platform for multiple disciplines and particularly used in STEM education. Most of the literature (75%) focus on university settings, with the majority (96%) on undergraduate studies. The bibliographic analysis shows the increasing trend in Moodle educational research and provides information about the top journals, leading authors, keywords, and high citations. The thematic analysis finds that Moodle is a powerful tool used to support learning in various ways. Both educators and students benefit from using the Moodle LMS, although currently at varying degrees. The most prevalent tools being used are Moodle “quizzes” and “workshops”, and external tools that can be easily embedded into the Moodle system are videos, virtual tours, and e-portfolios. Moodle enables the creativity of individual teachers to develop course-specific materials for students. In addition, Moodle saves time due to randomly generated tests, questions with multiple possible answers, automated marking systems and rubrics, and positive and motivational automatic summative and formative feedback. There is strong evidence that Moodle increases student engagement, performance, and satisfaction while enhancing flexibility in their learning environments. Areas showing a rapid growth in research are adaptive content and assessment development, improvements in data security, and user verification. Regardless of recent advancements in online teaching and learning, some studies report numerous fundamental gaps and drawbacks.

The gaps identified in this review are significant for future research. Some gaps include comparing Moodle with other LMSs and elaborating on the many e-learning tools and associated plug-ins available in the market but not analysed in educational research. Future research could focus on aspects pivotal for e-learning success: features, integration, cost, and security. Further research is needed to outline Moodle e-learning experiences in primary and secondary education settings, with qualitative studies needed, particularly focusing on teachers’ perspectives in a tertiary education setting. As only 5% of the studies have considered educational theories, future research needs to strengthen the theoretical underpinning of studies. Existing educational theories could successfully theorise the efficiency of content developments and the effectiveness of online study materials and assignments. Data gathering tools and statistical tools embedded into LMSs along with theoretical frameworks could lead to insightful research. As only 10% of articles discussed ethical aspects, more publications are needed to analyse ethical issues associated with e-learning, particularly focusing on the increasing number of artificial intelligence tools. More research on these aspects will help educators to utilise LMSs for successful online or blended course developments. As this review is based only on published articles, more applications of Moodle might be occurring, particularly in developing countries. Therefore, an area of future study could be a study examining statistics of Moodle usage rather than published papers.

## Data Availability

The data sets used and analysed during the current study are available from the corresponding author on request.
